# Clinical characteristics and serum HDL-C are associated with adverse prognosis in patients with acute decompensated heart failure with preserved ejection fraction at admission

**DOI:** 10.3389/fcvm.2026.1656574

**Published:** 2026-06-22

**Authors:** Xun Ma, Wen-bo Li

**Affiliations:** 1 Department of Emergency Internal Medicine, Shaanxi Provincial People’s Hospital, Shaanxi, China; 2 The Third Department of Cardiovascular Medicine, Shaanxi Provincial People’s Hospital, Shaanxi, China

**Keywords:** core symptom group, ejection fraction, heart failure, prognosis, serum lipid parameters

## Abstract

**Purpose:**

This study aimed to investigate whether clinical characteristics and lipid metabolism parameters are associated with adverse prognosis in patients hospitalized for acute decompensated heart failure (HF) with preserved or reduced left ventricular ejection fraction (LVEF).

**Methods:**

This study included 158 patients with chronic heart failure who were hospitalized at the Shaanxi Provincial People’s Hospital, Shaanxi, China, between January 2021 and October 2024. They were categorized based on their ejection fraction (EF) into a heart failure preserved EF group [heart failure with preserved ejection fraction (HFpEF); *n* = 73] and a heart failure reduced EF group [heart failure with reduced ejection fraction (HFrEF); *n* = 85]. The association of their clinical characteristics and lipid energy metabolism parameters with disease prognosis was assessed using univariate and multivariate logistic regression models. Univariate and multivariate logistic regression analyses were performed to identify independent predictors of adverse cardiovascular events. Receiver operating characteristic (ROC) curve analysis was performed to assess the independent prediction performance of the potential risk or protective factors.

**Results:**

The HFpEF and HFrEF groups showed significant differences in clinical symptoms, including palpitations/angina, cough, sleep at night, lack of appetite, weight loss, abdominal distension, worrying, irritability, decreased libido, and hypertension (*p* < 0.05). Furthermore, the groups showed significant differences in peak systolic BP, peak diastolic BP, total cholesterol, and high-density lipoprotein cholesterol (HDL-C) (*p* < 0.05). The multivariate logistic regression analysis results showed that a high level of HDL-C has certain discriminatory ability in the prognosis assessment of patients with heart failure (OR = 0.265, 95% CI: 0.085–0.781; *p* = 0.038), and the AUC value is 0.611 (95% CI: 0.519–0.703, *p* = 0.021).

**Conclusions:**

In patients admitted with acute decompensated HF, clinical indicators of congestion, psychological distress, blood pressure load, total cholesterol, and HDL-C are associated with adverse prognosis. Higher HDL-C shows modest but independent prognostic value in acute HF, especially in HFpEF. Further studies are warranted to explore dynamic changes in HDL-C and its role as a metabolic biomarker in HFpEF.

## 1 Introduction

Heart failure (HF) is a severe manifestation or terminal stage of various cardiovascular diseases (CVDs) and is associated with high disability and mortality rates. It is a complex clinical syndrome caused by impairments in the cardiac structure or function, thereby hindering ventricular filling or ejection function (EF) (1). Heart failure is classified into the following three categories based on left ventricular ejection fraction (LVEF): (i) heart failure with reduced ejection fraction (HFrEF), (ii) heart failure with mildly reduced ejection fraction (HFmrEF), and (iii) heart failure with preserved ejection fraction (HFpEF) (2). At present, targeting the renin–angiotensin–aldosterone system is the cornerstone of clinically improving hemodynamics and the prognosis of HF patients. However, traditional HF drugs such as angiotensin-converting enzyme inhibitors and β receptor blockers can reduce the hospitalization rate of patients with HFpEF but cannot improve the long-term prognosis of patients with HF. In recent years, myocardial substrate metabolism, particularly lipid and fatty acid utilization, has emerged as a promising therapeutic target for HF (3). However, the relationship between routine lipid profiles, clinical congestion symptoms, and short-term prognosis in acute decompensated HF remains incompletely defined, especially in HFpEF. Accordingly, this retrospective study analyzed clinical and laboratory data from patients admitted with acute decompensated HF to identify prognostic factors related to clinical presentation and lipid metabolism. The primary aim was to develop evidence to support risk stratification and targeted management of acute HF, particularly HFpEF.

## 2 Methods

### 2.1 Study subjects

This retrospective cohort study enrolled 158 patients hospitalized with acute decompensated chronic HF at Shaanxi Provincial People's Hospital between January 2021 and October 2024. The study procedure was approved by the ethics committee of the Shaanxi Provincial People's Hospital (2023-YBSF-619) and was conducted in accordance with the local legislation and institutional requirements. Informed consent was obtained from all the individual participants included in the study. The inclusion criteria were as follows: (i) patients who met the 2024 national guidelines for heart failure diagnosis criteria (4); (ii) age of 18 years old and above; (iii) sinus rhythm; (iv) New York Heart Association (NYHA) cardiac function grades II–IV; (v) patients who met the diagnostic criteria for HFmrEF in 2021 European Society of Cardiology (ESC) guidelines for the diagnosis and treatment of acute and chronic HF, including (a) clear symptoms and signs of HF, (b) LVEF ranging between 4l% and 49%, and (c) B-type natriuretic peptide (BNP) level of 35 pg/mL or N-terminal B-type B natriuretic peptide (NT-pro BNP) level of 125 pg/mL; and (vi) all patients included in the study received guideline-directed medical therapy for heart failure. The exclusion criteria were as follows: (i) complicated by malignancy or other acute critical disease, (ii) severe hepatic or renal insufficiency, and (iii) symptomatic bradycardia or second- or third-degree atrioventricular block without a pacemaker installation. Both patients and families were informed of the study protocol before the trial and signed the informed consent form.

### 2.2 Data collection

The extracted baseline data included demographic characteristics (age, gender, BMI, smoking, and alcohol use); clinical vulnerability syndrome [based on the Chinese version of the Memorial Heart Failure Symptom Assessment Scale with 20 symptom items, including 14 physical symptoms (PHYS), 3 psychological symptoms (PSYCH), and 3 heart failure symptoms (HFS)]; vital signs, including peak systolic and diastolic blood pressure; echocardiographic parameters [LVEF, left atrial diameter, left ventricular end-diastolic diameter (LVEDD), left ventricular end-systolic diameter (LVESD), and global longitudinal strain (GLS) where available]; NYHA classification; HF duration and type of HF; laboratory parameters, including complete blood count, coagulation, liver and renal function, lipid profile, and NT-proBNP; comorbidities, including hypertension, diabetes, coronary artery disease, arrhythmia, and valvular disease; and medications and device therapies.

### 2.3 Statistical methods

The statistical analysis of the data was performed using SPSS 26.0. Normally distributed measurement data are expressed as mean ± standard deviation (xˉ±s) and group comparisons were performed using the two-sample *t*-test. Non-normally distributed data are expressed as median (P25, P75) and group comparisons were performed using the Mann–Whitney *U*-test. The count data are expressed by the number of cases and percentage (%), and the group comparisons were performed using the chi-square test or Fisher’s exact test. *p* < 0.05 was considered statistically significant. Univariate logistic regression was used to screen potential prognostic factors. Variables with *p* < 0.05 were entered into the multivariate logistic regression to identify independent predictors of adverse cardiovascular events, defined as cardiovascular death or HF-related rehospitalization. ROC curve analysis was performed to assess the prognostic performance of significant biomarkers.

## 3 Results

### 3.1 Comparison of baseline data between the HFpEF and HFrEF groups

The comparison of baseline data between the HFpEF and HFrEF groups is shown in Table 1. There were no statistically significant differences between the two groups in terms of gender, mode of admission (emergency or outpatient), origin (rural/suburban), smoking status, drinking status, repeated hospitalization due to the same cause, NYHA classification, type of HF, presence of diabetes, presence of arrhythmia, presence of eight PHYS (nausea, numbness in hands or feet, paruria, vomiting, diarrhea, sweating, dizziness, and astriction), presence of one PSYCH (sleep disorder), and presence of one HFS (difficulty in breathing when lying flat) (*p* > 0.05). However, there were statistically significant differences between the two groups in terms of the presence of five PHYS (cough, abdominal distension, decreased libido or reduced sexual activity, loss of appetite, and loss of weight), presence of two PSYCH (worrying and irritability), presence of two HFS (heart palpitations/angina pectoris and awakening during sleep at night), and presence of hypertension (*p* < 0.05).

**Table 1:** Baseline data and comparison of clinical symptoms between the two groups.

Variable	Preserved left ventricular ejection fraction group (*n* = 73)	Reduced left ventricular ejection fraction group (*n* = 85)	*Χ*^2^/*Z*	*p*-value
Baseline information				
Outpatient/emergency	31/42	31/54	0.592	0.442
Residence (rural/suburban)	49/24	59/26	0.095	0.758
Gender (male/female)	34/39	44/41	0.423	0.515
Smoking, *n* (%)	13 (17.8%)	13 (15.3%)	0.181	0.671
Alcohol consumption, *n* (%)	2 (2.7%)	2 (2.4%)	–	1.000
Repeated hospitalization (same cause), *n* (%)	53 (72.6%)	61 (71.8%)	0.014	0.907
Echocardiographic parameters				
LVEF, %	56.2 ± 3.1	32.5 ± 4.6	12.34	0.000
Left atrial diameter, mm	45.8 ± 4.2	48.1 ± 5.3	2.87	0.005
LVEDD, mm	48.5 ± 3.6	62.3 ± 6.1	14.52	0.014
LVESD, mm	32.1 ± 2.9	49.6 ± 5.8	16.71	0.001
GLS, %	−18.2 ± 2.1	−10.5 ± 1.8	13.68	0.003
Physiological symptoms (PHYS)				
Cough, *n* (%)	35 (47.9%)	55 (64.7%)	4.500	0.034
Nausea, *n* (%)	9 (12.3%)	15 (17.6%)	0.862	0.353
Numbness in hands or feet, *n* (%)	7 (9.6%)	13 (15.3%)	1.156	0.282
Abdominal distension, *n* (%)	17 (23.3%)	37 (43.5%)	7.153	0.007
Paruria, *n* (%)	2 (2.7%)	6 (7.1%)	–	0.288
Vomiting, *n* (%)	4 (5.5%)	4 (4.7%)	–	1.000
Diarrhea, *n* (%)	5 (6.8%)	7 (8.2%)	0.107	0.743
Sweating, *n* (%)	4 (5.5%)	8 (9.4%)	0.865	0.352
Decreased libido/sexual activity, *n* (%)	12 (16.4%)	40 (47.1%)	16.677	0.000
Itchy skin, *n* (%)	5 (6.8%)	7 (8.2%)	0.107	0.743
Loss of appetite, *n* (%)	23 (31.5%)	55 (64.7%)	17.317	0.000
Dizziness, *n* (%)	28 (38.4%)	22 (25.9%)	2.825	0.093
Loss of weight, *n* (%)	21 (2 8.8%)	41 (48.2%)	6.243	0.012
Astriction, *n* (%)	12 (16.4%)	16 (18.8%)	0.153	0.695
Psychological symptoms (PSYCH)				
Difficulty falling asleep	24	38	2.852	0.240
Losing sleep	45	41		
Easy to fall asleep	4	6		
Worrying, *n* (%)	16 (21.9%)	38 (44.7%)	9.065	0.003
Irascibility, *n* (%)	4 (5.5%)	16 (20.0%)	6.325	0.012
Heart failure symptoms (HFS)				
Heart palpitations/angina pectoris, *n* (%)	66 (90.4%)	66 (77.6%)	4.654	0.031
Awakening during sleep at night, *n* (%)	8 (11.0%)	28 (32.9%)	10.787	0.001
Difficulty breathing when lying flat, *n* (%)	32 (43.8%)	44 (51.8%)	0.989	0.320
Complicating disease				
Hypertension, *n* (%)	61 (83.6%)	81 (95.3%)	5.940	0.015
Diabetes mellitus, *n* (%)	19 (26.0%)	27 (31.8%)	0.626	0.429
Arrhythmia, *n* (%)	67 (91.8%)	79 (92.9%)	0.075	0.784
NYHA classification				
Ⅱ level	3	2	6.486	0.078
Ⅱ–Ⅲ level	7	2		
III level	18	34		
IV level	6	7		
Type of heart failure				
Left heart failure	58	64	5.974	0.113
Right heart failure	3	9		
Whole heart failure	11	7		
Special type of heart failure, such as mitral stenosis	1	5		

### 3.2 Comparison of laboratory parameters between the HFpEF and HFrEF groups

As shown in Table 2, no significant differences were observed in weight, height, length of stay, routine blood tests, glucose, triglycerides, low-density lipoprotein cholesterol (LDL-C), or NT-proBNP (*p* > 0.05). Significant differences were identified in peak systolic blood pressure, peak diastolic blood pressure, total cholesterol, and high-density lipoprotein cholesterol (HDL-C) (*p* < 0.05).

**Table 2:** Comparison of laboratory results between the two patient groups.

Indicator	Preserved left ventricular ejection fraction group (*n* = 73)	Reduced left ventricular ejection fraction group (*n* = 85)	T/*Z*	*p*-value
General information				
Weight (kg)	59.75 ± 11.46	58.13 ± 11.43	0.886	0.377
Height (m)	1.63 (1.58, 1.71)	1.65 (1.60, 1.71)	−0.723	0.470
Age (year)	77.69 ± 9.38	78.18 ± 10.15	−1.318	0.187
Total duration of hospitalization (*d*)	6.85 ± 1.71	7.48 ± 2.55	−1.859	0.065
Maximum systolic blood pressure (mmHg)	140.00 (120.00, 159.00)	148.00 (130.00,1 80.00)	−2.386	0.017
Maximum diastolic blood pressure (mmHg)	85.00 (71.00, 98.50)	90.00 (79.00, 110.00)	−2.227	0.026
Peripheral blood cells				
White blood cells (4.3–11.3 × 10^9^/L)	6.06 ± 2.54	6.63 ± 2.38	−1.453	0.148
Hemoglobin (121–158 g/L)	111.30 ± 20.74	112.37 ± 26.88	0.773	0.441
Platelet count (PLT; 177–446 × 10^9^/L)	170.34 ± 56.12	176.09 ± 68.80	−0.570	0.570
Neutrophil granulocyte count (1.6–7.8 × 10^9^/L)	4.59 ± 2.25	4.67 ± 2.30	−0.226	0.822
Lymphocyte count (1.5–4.6 × 10^9^/L)	1.32 (0.73, 2.10)	1.10 (0.82, 1.53)	−1.704	0.088
Monocyte count (0.13–0.76 × 10^9^/L)	0.54 (0.44, 0.77)	0.61 (0.48, 0.75)	−0.978	0.328
Eosinophil count (0–0.68 × 10^9^/L)	0.10 (0.05, 0.17)	0.13 (0.06, 0.21)	−0.977	0.328
Neutrophil-to-lymphocyte ratio (0.88–4.00)	4.73 ± 3.80	5.46 ± 5.16	−1.287	0.200
Coagulation function				
D-dimer (0–0.55 mg/L)	2.31 (1.05, 5.96)	2.66 (1.02, 5.45)	−0.647	0.518
Prothrombin time (10.0–16.0 s)	14.30 (11.93, 18.06)	13.02 (11.74, 16.99)	−1.093	0.274
International normalized ratio of prothrombin (0.75–1.25)	1.27 (1.01, 1.56)	1. 11 (1.00, 1. 50)	−1.312	0.190
Activated partial thromboplastin time (20.0–40.0 s)	33.99 (30.82, 41.11)	35.53 (29.13, 41.65)	−0.479	0. 632
Thrombin time (14.0–21.0 s)	18.51 ± 1.24	18.41 ± 1.23	0.463	0.644
Fibrinogen (1.8–4.0 g/L)	3.66 ± 1.23	3.59 ± 1.36	0.331	0.741
Biochemical indexes				
Blood glucose (3.90–6.10 mmol/L)	6.31 (4.61, 7.79)	6.25 (4.63, 7.77)	−0.044	0.965
Triglycerides (0.45–1.69 mmol/L)	0.95 (0.59, 1.29)	1.06 (0.76, 1.40)	−1.602	0.109
Total cholesterol (3.00–5.20 mmol/L)	3.20 (2.83, 3.79)	3.70 (3.11, 4.49)	−2.578	0.010
Low-density lipoprotein cholesterol (LDL-C; 2.07–3.10 mmol/L)	2.16 ± 0.94	2.33 ± 0.98	−1.070	0.286
High-density lipoprotein cholesterol (adult male 1.16–1.42 mmol/L, adult female 1.29–1.55 mmol/L)	1.16 ± 0.33	1.03 ± 0.30	−2.459	0.015
Total protein (65.0–84.0 g/L)	60.21 ± 6.37	60.89 ± 7.15	−0.628	0.531
Albumin (39.0–54.0 g/L)	35.35 ± 4.32	34.35 ± 5.21	1.296	0.197
Alanine aminotransferase (5.0–40.0 U/L)	20.41 (12.00, 45.07)	19.00 (11.00, 54.32)	−0.140	0.888
Aspartate aminotransferase (14.0–44.0U/L)	26.20 (18.00, 52.33)	23.00 (17.00, 67.85)	−0.295	0.768
Creatine kinase isozyme (0–18.0 U/L)	3.86 (2.13, 6.13)	4.58 (1.90,9.00)	−1.443	0.149
Lactic acid (0.5–1.7 U/L)	1.48 ± 0.75	1.62 ± 0.72	−0.985	0.327
Serum procalcitonin (0∼0.5 ng/mL)	0.79 (0.07, 2.07)	0.70 (0.10,1.79)	−0.038	0.970
N-terminal pro-B-type natriuretic peptide (＜50 years: ＜125 pg/mL 50–75 years: ＜300 pg/mL ＞75 years: ＜900 pg/mL)	4,145.50 (1,756.88, 7,458.03)	4,696.63 (2,708.85 9,560.60)	−0.987	0.324

Data are expressed as mean ± standard deviation or median (interquartile range).

### 3.3 Predictors of adverse prognosis in acute HF

The univariate and multivariate logistic regression analyses are summarized in Table 3. After adjustment for confounding factors, higher HDL-C remained an independent protective factor for adverse cardiovascular events (OR = 0.265, 95% CI: 0.085–0.781; *p* = 0.038).

**Table 3:** Univariate and multivariate logistic regression analyses of factors associated with the prognosis of patients with preserved left ventricular ejection fraction.

Variable	*B*	SE	Wald	*p*	OR	95% CI
Physiology symptoms (PHYS)						
Cough	0.406	0.387	1.105	0.293	1.501	0.704–3.203
Lack of appetite	1.047	0.592	3.125	0.077	2.848	0.892–9.087
Loss of weight	0.094	0.478	0.039	0.844	1.098	0.431–2.801
Psychological symptoms (PSYCH)						
Worrying	−0.671	0.676	0.986	0.321	0.511	0.136–1.923
Irascibility	0.523	0.725	0.520	0.471	1.687	0.407–6.985
Heart failure symptoms (HFS)						
Palpitation and angina pectoris	−0.248	0.611	0.165	0.684	0.780	0.236–2.583
Paroxysmal nocturnal	0.778	0.676	1.323	0.250	2.177	0.578–8.197
Complicating disease						
Hypertension	1.194	0.744	2.575	0.109	3.301	0.768–14.192
General information						
Maximum systolic blood pressure (mmHg)	0.000	0.010	0.000	0.993	1.000	0.980–1.020
Biochemical indexes						
Total cholesterol (mmol/L)	0.272	0.189	2.078	0.149	1.313	0.907–1.902
High-density lipoprotein cholesterol (mmol/L)	1.184	0.616	3.697	0.038	0.265	0.085–0.781

B, regression coefficient; SE, standard error; OR, odds ratio; CI, confidence interval.

### 3.4 Prognostic performance of HDL-C

**Figure 1:**
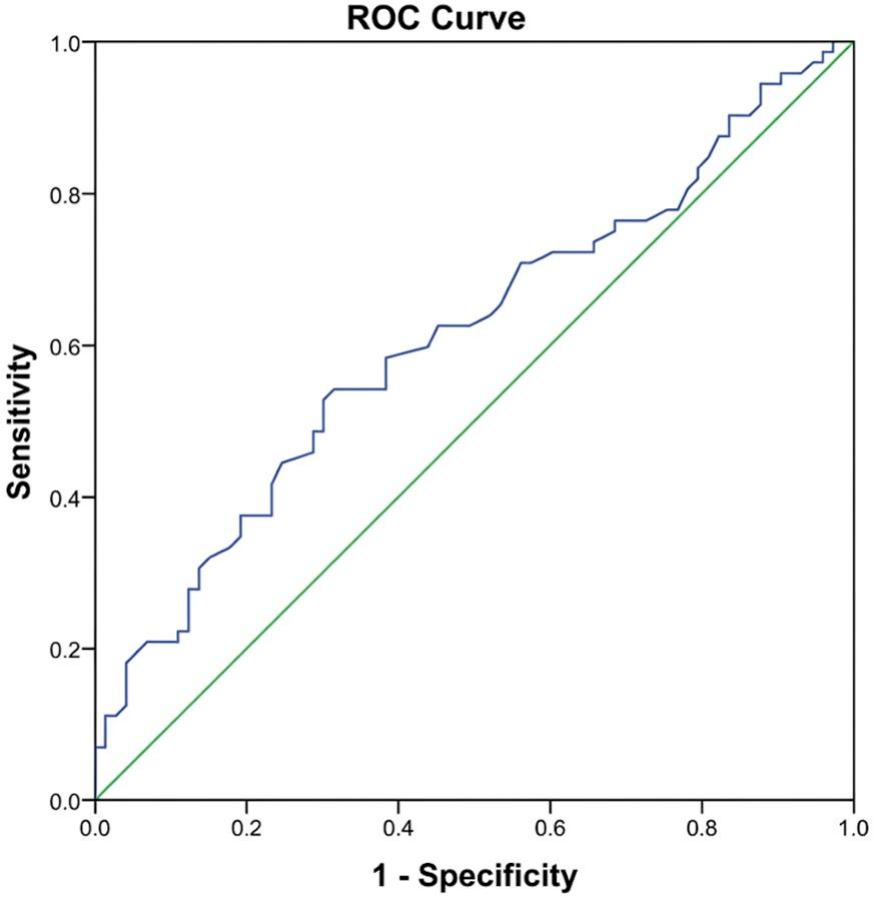
High levels of HDL-C predict a better prognosis in patients with heart failure.

ROC curves were drawn with the time of occurrence as the status variable (“0” = good quality of life, “1” = cardiovascular death and/or worsening of heart failure) and HDL-C prediction probability in the multivariate logistic regression analysis as the test variable. The results showed that the AUC value for elevated levels of HDL-C was 0.611 (95% CI: 0.519–0.703, *p* = 0.021) (Figure 1). At a cut-off value of 1.145, the sensitivity and specificity values for high-level HDL-C in the diagnostic model were 54.2% and 68.5%, respectively, and the Youden index was 0.227 (Table 4).

**Table 4:** Diagnostic performance of HDL-C for heart failure.

Variable	Cut-off	Sensitivity (%)	Specificity (%)	AUC (95% CI)	*p*
HDL-C	1.145	54.2	68.5	0.611 (0.519–0.703)	0.021

## 4 Discussion

HF is the end stage of many CVDs. According to epidemiological surveys, the number of patients with HF is estimated to be more than 64.4 million by the year 2030 (5). According to the 2021 ESC Guidelines for the Treatment of Acute and Chronic HF (2), the three categories of patients with HF based on their different degrees of ejection fraction, namely, HFrEF (LVEF: ≤40%), HFmrEF (LVEF: 41%–49%), and HFpEF (LVEF: ≥50%), have distinct clinical phenotypes and prognostic characteristics (6). HFpEF accounts for approximately 50% of all patients with HF and is one of the main types of HF (7, 8). The China Hypertension Survey results showed that the prevalence of HF was 1.3% in Chinese adults aged 35 years and older, accounting for nearly 8.9 million patients with HF in China; HFpEF accounts for 36% of this HF population and is increasing at a rate of 1% per year (9). In this study, the prevalence of HFpEF was 46.2%. This estimate is slightly higher than the previous report, probably because the cohort in our study included a more elderly population with other underlying diseases such as hypertension, diabetes, and coronary arteriosclerosis. Several studies have confirmed that comorbidities such as diabetes and hypertension are the main risk factors for cardiovascular and cerebrovascular diseases. Since these comorbidities are closely related to the prevalence of HFpEF, this study cohort showed a higher prevalence of HFpEF. However, the prevalence of HFpEF is expected to be even higher after accounting for population aging and advances in medical treatments. Since the causes of HF are heterogeneous, early detection of abnormal heart conditions can lead to early diagnosis and treatment of HF. In such cases, understanding the clinical and metabolic characteristics and the core symptoms of patients with HFpEF at the hospital will enable better clinical treatment and prognosis evaluation for such patients, thereby improving their overall prognosis.

Patients with acute HF experience a complex symptom cluster reflecting pulmonary and peripheral congestion, volume overload, and heightened sympathetic activation (10, 11). Multiple studies (12–14) have shown that compared with a single symptom, multiple symptoms synergistically increase the disease burden in patients with HF, reduce their treatment compliance, and significantly impact their quality of life and health status. Symptom cluster management is a domestic and foreign research hotspot and a significant new direction for the management of HF symptoms. However, the majority of domestic and foreign research studies on HF symptoms are cross-sectional and overlook core symptoms in the early stages of hospitalization. Our study identified significant phenotypic differences between HFpEF and HFrEF in cough, abdominal distension, anorexia, anxiety, and irritability. These between-group phenotypic differences highlight distinct clinical patterns in HF subtypes. These symptoms likely reflect varying degrees of congestion and tissue hypoperfusion, which in turn contribute to worse functional status and prognosis. During acute admission, relieving pulmonary congestion and improving dyspnea should be prioritized to stabilize clinical status and prevent early deterioration. These findings are consistent with the findings of Jurgens et al. (15). However, compared with the study by Xuan et al. (16), our study included more symptoms. This suggests that the symptom group extracted in this study could better reflect the variability in the characteristics observed in patients with acute heart failure, including reduced heart output, insufficient blood perfusion to organs and tissues, accelerated compensatory heart rate, palpitations and fatigue, and symptoms of heart and tissue ischemia. EF decompensation leads to increased congestion of peripheral and pulmonary tissues, leading to significant congestion of the digestive tract, abdominal distension, and decreased appetite. Pulmonary congestion causes cough and disturbed sleep at night, leading to psychological symptoms, such as anxiety and irritability, and their aggravation. Interactions between each syndrome lead to HF aggravation and poor prognosis. Furthermore, compared with previous studies, the types of symptom clusters were different in our study. This may be due to inconsistent statistical methods (17, 18), the survey assessment tools used (19), and the number of symptoms included (20). However, based on the overall research results, during the acute admission period, symptoms related to dyspnea are accompanied by psychological and emotional symptoms. Therefore, in the acute patient admission phase, medical staff should take measures to mitigate dyspnea caused by pulmonary congestion. This will be beneficial for controlling heart failure during HFpEF, facilitating effective standardized management of the disease, and improving the prognosis.

NT-proBNP is widely recognized as a biomarker of wall stress and congestion in HF (21, 22). In the present study, NT-proBNP levels did not differ significantly between the HFpEF and HFrEF groups, possibly because both groups presented with acute decompensation and comparable volume overload. Additionally, medication use, including angiotensin receptor neprilysin inhibitors, may have modulated natriuretic peptide levels. These findings highlight the need for caution when interpreting NT-proBNP levels solely for phenotypic discrimination in acute HF. Several studies (23, 24) have shown that plasma NT-proBNP levels are higher in women than in men, which may be related to differences in hormone levels. NT-proBNP is mainly cleared and excreted by the kidneys and other organs. Therefore, renal insufficiency affects plasma NT-proBNP levels (25). Previous studies (26) have confirmed that atrial expansion, increased serum ion concentration, increased angiotensin, and other diseases in patients with arrhythmia, such as atrial fibrillation and HF, can stimulate the heart to release NT-proBNP. Furthermore, studies (27) have confirmed that some drugs can influence NT-proBNP plasma levels. For example, when drugs containing neutral endopeptidase inhibitors, such as sacubitril/valsartan, are used to treat HF, degradation of BNP is reduced. In this study, both patient groups received the aforementioned drug treatments, but subgroup medication data are lacking. Thus, we can only speculate that changes in NT-proBNP levels are influenced by drug interactions. Studies with larger sample sizes and subgroup drug analyses are still needed to further clarify the correlation.

Peak systolic and diastolic blood pressure differed significantly between the groups, consistent with altered vascular compliance and pressure overload commonly seen in HFpEF. Blood pressure parameters may help support phenotypic diagnosis using established scores, such as H2FPEF and HFA-PEFF, which are easily applicable in routine clinical settings (28, 29).

Lipid metabolism plays a critical role in myocardial function, inflammation, and oxidative stress in HF (30). Lower HDL-C has been linked to coronary microvascular dysfunction, endothelial impairment, and worse cardiovascular outcomes. In our analysis, higher HDL-C was independently associated with a favorable prognosis, likely through reverse cholesterol transport and anti-inflammatory and anti-atherogenic effects. Although the AUC value indicates modest discriminative ability, HDL-C may still serve as a convenient metabolic biomarker for risk stratification in acute HF. This value reflects its limited discriminatory ability, and the external validity is limited by the single-center, retrospective design and small sample size. Several studies have shown that hyperlipidemia is related to various pathological cardiac metabolic mechanisms, including myocardial fibrosis, oxidative stress, inflammation, and endothelial dysfunction, all of which promote the progression of HFpEF (31). Multiple studies have confirmed that reduced HDL-C level is an important driver of non-stenotic coronary microcirculation disorder in patients with coronary heart disease (31, 32). Several studies have confirmed that the incidence of HFpEF is increasing with a rising prevalence of hyperlipidemia and hyperuricemia. Hyperlipidemia promotes cardiac oxidative stress and inflammation and alters cardiac metabolism, especially fatty acid metabolism, and subsequently exacerbates HFpEF pathology (33). HDL-C is involved in reverse cholesterol transport and is associated with anti-inflammatory and anti-atherosclerosis effects. Therefore, it reduces blood cholesterol levels and, to a certain extent, decreases hyperlipidemia, thereby improving local lesions and the function of coronary vascular smooth muscle cells and endothelial cells, and increasing coronary blood flow reserve, myocardial blood flow and capillary density, and myocardial circulatory perfusion. Through these mechanisms, it regulates myocardial oxygen levels and nutrient and metabolite exchange, thereby effectively improving the prognosis of HFpEF. Therefore, HDL-C is a target for treating heart failure and is a potential indicator of the progression and prognosis of HFpEF (34). It should be noted that our study is merely a retrospective analysis of conventional serum lipid parameters, and there is still a lack of data regarding myocardial energy metabolism. In the future, we plan to further integrate the analysis of relevant basic experiments with clinical data to provide more effective research evidence.

Although there are studies supporting HDL-C as a protective factor in cardiovascular diseases, the specific mechanism of its action in patients with HFpEF has not yet been identified. A few studies have suggested that HDL-C affects the progression of heart failure by regulating lipid metabolism and improving insulin sensitivity (35). However, other studies have suggested that the dysfunction associated with lower HDL-C levels, including reduced cholesterol clearance, may be associated with poor prognosis in heart failure (36). Low HDL-C levels are directly associated with an increased incidence of cardiovascular events (37). This phenomenon may be related to the dual role of HDL in lipid metabolism and inflammation regulation. Although low HDL-C is closely related to the progression of arteriosclerosis and cardiac metabolic disorders, the specific mechanism of HDL-C in patients with HFpEF still needs further exploration. Therefore, although the findings of this study are consistent with the existing literature, the mechanism of action of HDL-C still needs to be further investigated, especially in patients with HFpEF.

This study demonstrates that in patients admitted with acute decompensated HF, multiple clinical features of congestion, hemodynamic load, and lipid metabolism are associated with adverse prognosis. Notably, higher HDL-C is independently associated with better outcomes, supporting its potential role as a prognostic biomarker in acute HF, especially in HFpEF.

Several limitations should be acknowledged. First, this was a single-center retrospective study with a relatively small sample, which may limit generalizability. Second, psychological symptoms were assessed during acute decompensation, which may be influenced by hemodynamic instability and thus should be interpreted cautiously. Third, we only evaluated routine lipid parameters rather than direct measures of myocardial energy metabolism. Finally, the lack of significant age and gender differences between the groups may reflect local referral patterns and should be interpreted with caution compared with large epidemiological studies.

## 5 Conclusions

In patients admitted with acute decompensated heart failure, clinical indicators of congestion, psychological distress, blood pressure load, total cholesterol, and HDL-C are associated with adverse cardiovascular outcomes. Higher HDL-C provides modest but independent prognostic value in acute HF, particularly in HFpEF. Phenotypic differences between patients with HFpEF and HFrEF are characterized by distinct symptom profiles and hemodynamic profiles. Future multicenter prospective studies are warranted to validate HDL-C as a dynamic metabolic biomarker and to explore targeted lipid-modulating strategies in acute decompensated HF.

## Data Availability

The original contributions presented in the study are included in the article/Supplementary Material, further inquiries can be directed to the corresponding author/s.
